# Sentinel Node Mapping in Non-small Cell Lung Cancer Using an Intraoperative Radiotracer Technique

**DOI:** 10.22038/AOJNMB.2019.13195

**Published:** 2019

**Authors:** Susan Shafiei, Reza Bagheri, Ramin Sadeghi, Vahid Reza Dabbagh Kakhki, Amir Hossein Jafarian, Reza Afghani, Davood Attaran, Reza Basiri, Shahrzad M Lari

**Affiliations:** 1 Nuclear Medicine Research Center, Mashhad University of Medical Sciences, Mashhad, Iran; 2 Lung Diseases Research Center, Mashhad University of Medical Sciences, Mashhad, Iran; 3 Cancer Molecular Pathology Research Center, Ghaem Hospital, School of Medicine, Mashhad University of Medical Sciences, Mashhad, Iran; 4 Thoracic Surgeon, Department of General Surgery, Azar Hospital, Golestan University of Medical Science, Gorgan, Iran; 5 Lung Disease Research Center, Faculty of Medicine, Mashhad University of Medicine Sciences, Mashhad, Iran

**Keywords:** Non-small cell lung cancer, Radiotracer, Sentinel node

## Abstract

**Objective(s):**

Lymph node metastases are the most significant prognostic factor in localized non-small cell lung cancer (NSCLC). Identification of the first nodal drainage site (sentinel node) may improve detection of metastatic nodes. Extended surgeries, such as lobectomy or pneumonectomy with lymph node dissection, are among the therapeutic options of higher acceptability. Sentinel node biopsy can be an alternative approach to less invasive surgeries. The current study was conducted to evaluate the accuracy of sentinel node mapping in patients with NSCLC using an intraoperative radiotracer techniques.

**Methods:**

This prospective study was conducted on 21 patients with biopsy-proven NSCLC who were candidates for sentinel node mapping during 2012-2014. All patients underwent thoracoabdominal computed tomography, based on which they had no lymph node involvement. Immediately after thoracotomy and before mobilizing the tumor, peritumoral injection of 2mCi/0.4 mL Tc-99m- phytate was performed in 4 corners of tumor. After mobilization of the tumoral tissues, the sentinel nodes were searched for in the hillar and mediastinal areas using hand-held gamma probe . Any lymph node with in vivo count twice the background was considered as sentinel node and removed and sent for frozen section evaluation. All dissected nodes were evaluated by step sectioning and hematoxylin and eosin staining (H&E).The recorded data included age, gender, kind of pathology, site of lesion, number of dissected sentinel nodes, number of sentinel nodes, and site of sentinel nodes. Data analysis was performed in SPSS software (version 22).

**Results:**

The mean age of the patients was 58.52±11.46 years with a male to female ratio of 15/6. The left lower lobe was the most commonly affected site (30.09%). Squamous cell carcinoma and adenocarcinoma were detected in 11 and 10 subjects, respectively. A total of 120 lymph nodes were harvested with the mean number of 5.71±2.9 lymph nodes per patient. At least one sentinel node was identified in each patient, resulting in a detection rate of 95.2%. The mean number of sentinel nodes per patient was 3.61±2. Frozen section results showed 100% concordance with the results of hematoxylin and eosin staining.

**Conclusion:**

Based on the findings, sentinel node mapping can be considered feasible and accurate for lymph node staging and NSCLC treatment.

## Introduction

Lung cancer is the most common cause of cancer-related mortality all over the world (1). Surgery is an integral component of the therapeutic course for this cancer, especially for non-small cell lung cancer (NSCLC). Lymph node status is the main predictor of treatment outcome and patients’ survival in this disease (1, 2). Given the special characteristics of lung cancer, extended surgeries, such as mediastinal lymph node dissection and lobectomy or pneumonectomy, are the routine surgical methods for this tumor (2). 

Only 20-25% of the clinically stage I NSCLCs are accompanied by mediastinal lymph node involvement and mediastinal lymph node dissection is considered highly invasive (2-4). Due to this fact, Several diagnostic methods have been described for the pre-operative diagnosis of mediastinal lymph node involvement, such as computed tomography (CT) scan, magnetic resonance imaging (MRI), 18F-fluorodeoxyglucose positron emission tomography, mediastinoscopy, and sentinel node mapping (2,5). 

Sentinel node mapping by means of radioisotopes can be an excellent method for the pathological evaluation of pathological lymph nodes status by removing a limited number of lymph nodes. This procedure guides the surgeon to omit regional lymph node dissection in patients with negative sentinel nodes (6,7). This method has been used for the management of many solid tumors, such as melanoma, breast, gastrointestinal, and genitourinary tumors (8,9). It has been also used for esophageal cancers, along with mediastinal lymph node dissection (10,11). 

The sentinel node is described as the first node in the lymphatic drainage pathways of tumors. The exact identification of the sentinel node permits the pathologists to concentrate on examinations and assists the surgeon in performing a complete nodal dissection (5,12). The potential role of sentinel node evaluation in limiting mediastinal node dissection was first reported in NSCLC patients in 1999. However, the exact role of this examination in the management of NSCLC remains to be determined (2,5). With this background in mind, the current study was conducted to investigate the feasibility of sentinel node mapping of NSCLC using an intraoperative radiotracer technique.

**Table 1:** Demographic and clinical data of patients

**Number of patients**	**21**
Age	58.52±11.46
Gender	
Male Female	15 6
Location of the tumor	
RMB RLL RUL RML LMB LLL LUL	1 4 3 1 1 8 3
Number of sentinel nodes per patient	
Median Mean	3 3.61±2
Number of dissected nodes per patient	
Median Mean	5 5.71±2.9
Location of the sentinel nodes	
Hilar Station 8 Station 9 Station 5 Station 7 Station 10 Interlobar Peri-bronchial	40 12 5 1 1 1 3 3

LMB: left main bronchus, RMB: right main bronchus, LLL: left lower lobe, RLL: right lower lobe, LUL: left upper lobe

**Table 2:** Results of sentinel node mapping in the patients

**No**	**Age**	**Gender**	**Tumor site**	**Pathology**	**Location of the SNs and their pathological involvement by frozen section/ H&E**								**Number of** **harvested** **SLN**	**Number of** **harvested** **non-SLN**	**Pathological** **involvement** **of the non-** **SLN**	**Total number** **of involved** **lymph nodes**
					**hilar **	**St 8**	**St 9**	**St 5**	**St 7**	**St 10**	**Interlobar**	**Peri-bronchial**				
**1**	**42**	**F**	LMB	SCC	1neg		1neg						2	1	0	0
**2**	**63**	**M**	RLL	SCC	2neg	1pos	2neg				3neg		8	1	1	2
**3**	80	F	LLL	AC		3pos		1pos					4	1	0	4
**4**	**59**	**M**	LLL	AC	2neg	2neg	1neg						5	6	0	
**5**	57	M	LUL	SCC	4neg								4	3	0	
**6**	**51**	**M**	RMB	SCC			1neg		1neg				2	2	0	
**7**	70	M	LLL	SCC	4pos								4	2	0	4
**8**	**58**	**M**	LUL	AC	2neg							3neg	5	6	0	
**9**	51	M	LUL	SCC	2neg	1neg							3	2	0	
**10**	**59**	**M**	LLL	SCC	1neg 1pos	1neg							3	1	0	1
**11**	67	M	LLL	AC	7neg								7	4	0	
**12**	**72**	**M**	RUL	SCC	4neg								4	1	0	
**13**	55	F	RLL	SCC	1neg 2pos	4neg							7	1	0	2
**14**	74	M	RLL	AC	1pos								1	2	0	1
**15**	57	M	RML	AC	0								0	1	0	
**16**	53	F	RLL	AC						1neg			1	1	0	
**17**	65	M	LLL	AC	3neg								3	1	0	
**18**	69	M	RUL	AC					2neg			3neg	5	2	0	0
**19**	44	F	RUL	AC		1pos						1pos	2	2	2	2
**20**	33	F	LLL	SCC	3neg								3	2	0	0
**21**	50	M	LLL	SCC	3neg								3	2	0	0

SLN: sentinel lymph node, H&N: hematoxylin and eosin, LMB: left main bronchus, RMB: right main bronchus, LLL: left lower lobe, RLL: right lower lobe, LUL: left upper lobe, RUL: right upper lobe, RML: right middle lobe, SCC: squamous cell carcinoma, AC: adenocarcinoma

**Figure 1:**
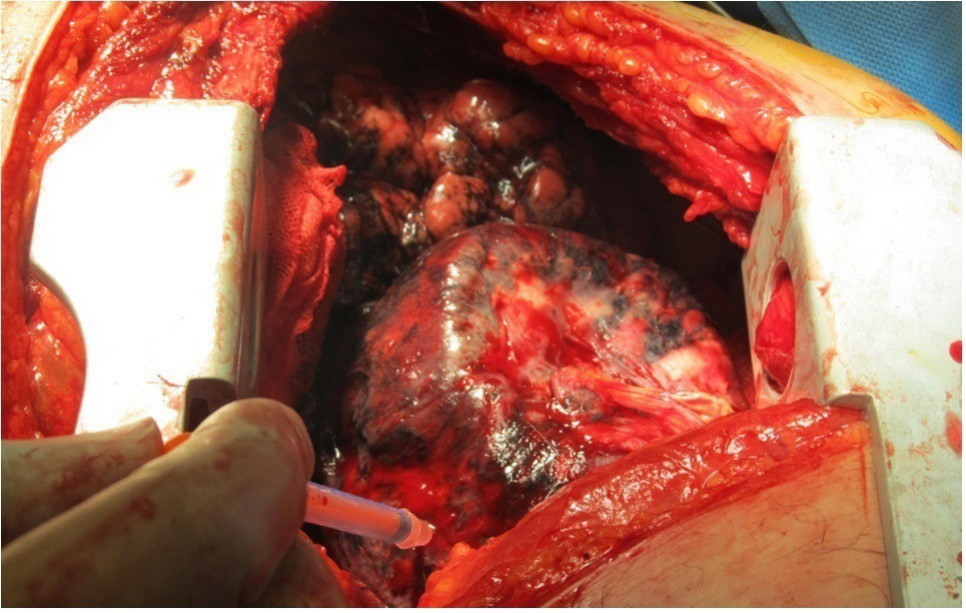
Non-small cell lung cancer after lung exposure and tracer injection in the peritumoral area

## Methods

This prospective study was conducted on 21 patients with biopsy-proven NSCLC who were candidates for sentinel node mapping from September 2012 to March 2014, 21. The NSCLC was diagnosed in patients based on transbronchial biopsy or transthoracic needle biopsy. All patients underwent thoracoa-bdominal CT scan, based on which no lymph node involvement was detected in any of the patients. Given the probability of inducing changes in the pattern of mediastinal lymph nodes after undergoing mediastinoscopy, this procedure was not performed in the current study. 

The patients with a history of neoadjuvant treatment and related comorbid states were excluded from the study. All patients underwent lobectomy or pneumonectomy via a right or left thoracotomy, followed by mediastinal lymphadenectomy. This study was approved by the Local Ethics Committee of Mashhad University of Medical Sciences, Mashhad, Iran. Furthermore, written informed consent was obtained from all participants before enrollment in the study.

***Intraoperative sentinel node mapping***

Immediately after thoracotomy, 1 mCi (0.4 ml) Tc-99 m-phytate was injected around the tumor in four divided specimens. Blue dye was not injected due to black discoloration of the mediastinal lymph nodes in most patients which can make it difficult to find dye-stained SNs.(3,13). Tc-99m-phytate is a radiopharmaceutical agent, which converts to particles with an average size of 150 nm upon reacting with the tissue calcium ion (6,14). In addition to regional lymph nodes, and reticuloendothelial system, biodistribution of Tc-99m-phytate includes urinary system (14), and growth plates (15). Tc-99m-phytate has been used for sentinel node mapping in various solid tumors with excellent results (16-21). 

Blue dye was not injected due to the black staining of the mediastinal lymph nodes in most of our patients which can complicate the finding of dye-stained sentinel nodes (2,12). After the mobilization of the mass and related lobes of the lung, taking 20-30 min on average, the sentinel nodes were explored in the hilar and mediastinal parts by means of a hand-held gamma probe (GPS Navigator, Tyco Healthcare, Tokyo, Japan). 

Any lymph nodes with an in vivo count twice that of the background was considered as a sentinel node and dissected, and then sent for frozen section evaluation (22-24). Background activity was detected by 10-second counting of the contralateral lung without the radiotracer injection (Figure 1). In the next stage, hilar and mediastinal lymphadenectomy was performed for all patients. All dissected nodes were assessed by step sectioning and hematoxylin and eosin staining. Sentinel nodes were also evaluated by frozen section. Same pathologist did the pathological examination of the sentinel and non-sentinel nodes in a non-blinded fashion.

***Statistical analysis***

The variables investigated in the present study included age, gender, type of pathology, site of lesion, number of dissected sentinel nodes, and number and site of sentinel nodes. This study also involved the evaluation of two indices, namely detection rate and false negative rate. The detection rate was described as the ratio of patients who had at least one detected sentinel node to the total number of patients. In addition, the false negative rate was the ratio of false negative sentinel nodes to all patients with involved lymph nodes with at least one harvested sentinel node (26).

## Results

In this prospective analysis, 21 patients (including 15 males and 6 females) with biopsy-proven NSCLC were selected for sentinel node mapping. The mean age of the participants was 58.52±11.46 years. The left lower lobe was the most commonly affected site (n=8, 30.09%). Furthermore, squamous cell carcinoma and

adenocarcinoma were detected in 11 (52.38%) and 10 (47.61%) patients, respectively. Every patient had at least one sentinel node, except for one patient with the adenocarcinoma of the right middle lobe, resulting in a detection rate of 95.2%. The mean and median numbers of the sentinel nodes per patient were 3.61±2 and 3, respectively. 

Table 1 presents the demographic characteristics of the patients and frequency distribution of the sentinel nodes. The mean number of the dissected nodes per patient was 5.71±2.9. Based on the findings, 7 patients had pathological lymph node involvement; in addition, the sentinel node was pathologically positive in all of the patients, rendering a false negative rate of 0%. The sentinel nodes were the only involved nodes among the dissected nodes. In one patient with adenocarcinoma of the right lung (middle lobe), no sentinel node was detected. Frozen section results showed 100% concordance with the results of hematoxylin and eosin staining (Table 2).

## Discussion

Lung cancer is in the list of the most important causes of cancer-related death in Iran similar to that in other Asian countries, as well as modern countries, such as US and Canada (14,25). The rate of lung cancer mortality is lower among Asian women than among the inhabitants of other western countries. However, this rate has been on an increasing trend in recent years (25). Surgery remains an important part of curative lung cancer therapeutic procedure, and lymph node dissection cannot be abbreviated in this approach due to its significant correlation with tumor staging, which is a reliable prognostic criterion (1, 5). 

For the majority of patients with early-stage NSCLC, mediastinal lymph node dissection and sampling not only will not lead to a survival benefit but also might increase the morbidity rate (2, 15). In this regard, sentinel node mapping via various tracers, such as blue dye or radioisotope tracers, has been suggested to be an effective method in reducing the possibility of lymph node dissection through the identification of the pathological sentinel node, metastases and accurate staging of solid tumors (2,8,10,15). 

Although the importance and reliability of sentinel node mapping procedure have been documented for breast cancer, there is insufficient evidence regarding the validity of this approach for NSCLC. The current study was targeted toward the evaluation of the accuracy of intraoperative peritumoral radiotracer injection for sentinel node mapping in patients with NSCLC. Given the high speed of radiotracer movement in the lymphatics (30,31), this procedure resulted in a high identification rate in the present study, with only one detection failure (6,16,17). 

Based on the evidence, sentinel node mapping in NSCLC is the most advantageous procedure for patients with small tumor size and no lymph node involvement based on initial clinical and radiological examinations (2,18). In the current study, all the included patients were lymph node negative as indicated by the preoperative examinations. This can be the reason for obtaining a very high detection rate and sensitivity. One patient with the adenocarcinoma of the right middle lobe (i.e., a hilar tumor) had no detectable sentinel lymph node, which might be due to the inability of in vivo counting in hilar lymph node stations to identify sentinel node due to the shine-through effect caused by the tumor radioactivity (20,21). 

In the current study, no blue dye was applied for intraoperative sentinel node mapping. In the same vein, the previous studies have reported difficulty in localization due to the black staining of the mediastinal lymph nodes in most of the patients (2,10,11,21). Hypersensitivity (even anaphylactic) reactions, pulse oximetry disturbances, and skin complications have been also reported as possible associated obstacles when using the different types of blue dyes for patients with melanoma or breast cancer (22,23). 

Our study revealed a high sentinel node detection rate (95.2%) by using radiotracer Tc-99m-phytate alone. Our result is consistent with previous reports on NSCLC, melanoma, and breast cancer, which revealed the significant accuracy, efficacy, and feasibility of intraoperative sentinel node mapping method by injecting radiotracer alone (16,24). A more important index of success in sentinel node mapping is sensitivity or false negative rate. No false negative case was observed in the present study, resulting in a sensitivity rate of 100%. This finding is in line with those of the previous studies reporting a low false negative rate and high accuracy rate for intraoperative radiotracer application in the sentinel node mapping of NSCLC (14,18,24).

Intraoperative evaluation of sentinel nodes is an important aspect of lymphatic mapping. In this regard, this procedure is essential for the omission of lymphadenectomy in the patients without pathological sentinel node involvement. In the current study, frozen section evaluation was used to assess the status of sentinel lymph nodes. The accuracy of frozen section was obtained at 100% given the complete concordance between the results of this evaluation and those of permanent hematoxylin and eosin staining. This was also in accordance with the results of the previous studies investigating sentinel node mapping in various malignancies (26).

***Skip pattern of lymphatic drainage and skip metastasis***

The skip metastasis is an important concept in NSCLC surgery. Mediastinal lymph node metastasis in the absence of hilar or interlobar lymph node metastasis is considered skip metastasis and can affect surgical planning (28,29). In our study, one patient (i.e., patient No. 3) showed skip metastasis in the mediastinal lymph nodes of stations 5 and 8 without hilar lymph node involvement. In two patients (i.e., patients No. 6 and 16), skip pattern of lymphatic drainage was also noted : sentinel lymph node localization in the mediastinal region without any hilar sentinel nodes. This indicates that a negative N1 station does not guarantee a tumor-free mediastinum. 

In a meta-analysis performed by Taghizadeh et al., skip metastases were reported in 18 studies out of the 41 included studies which is in line with our results (2). This fact, as well as the high sensitivity of sentinel node mapping for the detection of N2 involvement, makes lymphatic mapping a useful method for the prediction of mediastinal lymph node involvement and skip metastasis. Lymphatic drainage can be also multi-directional, thereby resulting in multiple sentinel nodes in different basins. Multiple sentinel nodes can also be found in a single basin, and all hot nodes should be harvested in order to maximize sensitivity (23). 

In our study, a lymph node with more than a two-fold activity than that of the background was regarded as sentinel node which could decrease the false negative rate. It should be mentioned that several new methods have been applied for sentinel node mapping in solid tumors, including NSCLC. Near-infrared fluorescent light and colloidal supermagnetic iron particles are among these upcoming modalities in NSCLC sentinel node mapping. Future studies should clarify the role of these modalities in more detail.

***Complications***

In our study, no significant complication related to lymphatic mapping was detected. However, various complications have been reported in previous studies as the consequences of the preoperative injection of tracers for sentinel node mapping in NSCLC. These complications include pneumothorax, bleeding, potential pleural tumor seeding, as well as requirement of CT-guided injection and skin marking before surgery which may be time consuming for patient and physician (and should be done almost 18 hours before surgery) (3,29-31). However, further studies are still needed to evaluate this finding in more details.

***Research Limitations***

Our study was limited in several aspects. First, the sample size was relatively small, thereby limiting the investigation of the relationships between tumor characteristics and sentinel node mapping performance. Another limitation was the non-implementation of positron emission tomography/CT evaluation before patient inclusion. More complimentary studies can be effective in increasing the 5-year survival rate. 

## Conclusion

As the findings of the present study indicated, the intraoperative peritumoral injection of the radiotracer facilitates lymphatic mapping and sentinel node biopsy with high detection rate and sensitivity. 
